# A conceptual model for evaluating eco-efficiency of thermal spraying processes

**DOI:** 10.1016/j.heliyon.2024.e32414

**Published:** 2024-06-04

**Authors:** Maria Julia Xavier Belem, Milton Vieira Junior, Giovanni Mummolo, Francesco Facchini

**Affiliations:** a Production Engineering Graduate Program, Methodist University of Piracicaba, Piracicaba, 13400-390, Brazil; b Ionian Department in Juridical and Economic Systems of the Mediterranean: Society, Environment, Cultures, University of Bari, 74100, Taranto, Italy; c Department of Mechanics, Mathematics and Management, Polytechnic University of Bari, 70125, Bari, Italy; d Universidade Presbiteriana Mackenzie, R. da Consolação, Consolação, São Paulo, 01302-907, Brazil

**Keywords:** Thermal spray, Eco-efficiency, Assessment model, Environmental indicators

## Abstract

Thermal spraying (TS) is one of the main processes for obtaining surfaces with the desired protective properties in various industrial applications. TS is an energy-intensive treatment required to heat the application material and consumes different resources. To assess the environmental impact of TS, it becomes necessary to integrate an approach that jointly analyses and evaluates the economic and environmental variables influencing the system. The concept of eco-efficiency (EE) added to the TS process allows for assessing the environmental and economic condition through the survey and application of eco-indicators. The lack of an EE evaluation model for TS processes was identified based on literature searches. Thus, the overall objective of this work is to propose a conceptual model to evaluate the EE of TS treatment, selecting environmental and economic indicators considered more impactful in the process. The model developed consists of three main steps: (i) the input and output indicators (environmental and economic) are identified by applying the Analytic Hierarchy Process (AHP) method; (ii) the structure to be employed in the model is defined; and (iii) the Data Envelopment Analysis (DEA) model is applied to define the EE evaluation form. The proposed model consists of clear and easy-to-follow steps for evaluating the EE of spraying processes, filling the gap found in the literature. The use of DEA allowed the integration of the environmental and economic indicators obtained from the TS processes to generate important insights for evaluating EE. The results prove the model's effectiveness in identifying the EE results for each analysed unit of the TS process. The model has provided an evaluation consistent with the existing studies, and the EE scores were assessed according to twenty-one decision-making units (DMUs) allowing the identification of the most eco-efficient DMUs concerning TS processes.

## 1 Introduction

The coating application process is an additional step in the manufacturing process, which involves the use of various materials and energy resources needed to ensure that the coatings offer benefits greater than their application cost. This justifies the importance of using a coating deposition process that meets the desired quality and resistance conditions. These protective coatings can be obtained through processes such as sol-gel, physical vapour deposition (PVD), chemical vapour deposition (CVD), welding (cladding), laser deposition and thermal spraying (TS) [1]. Deposition technologies such as PVD and CVD require a controlled pressure environment to obtain a thin, highly resistant film. Generally, these processes are expensive and have limits regarding the size and shape of the part to be coated [2].

However, TS treatment has several advantages compared to most coating application processes, such as lower process cost, higher production rate [1,3], and greater variety in terms of materials that can be applied, layer thicknesses and possible coating protection characteristics [4,5]. Furthermore, although this process has a non-negligible environmental impact, it is less impactful compared to other known corrosion resistance processes, such as chrome plating. In this way, TS can be used to replace chrome plating, which produces emissions of hexavalent chromium, a carcinogen and, therefore undesirable to use, i.e., harmful to health and the environment [6].

Spraying processes use different temperatures and deposition speeds. The processes that are known for having a higher velocity in their spray jet, referred to as supersonic, are high-velocity oxy-fuel coating (HVOF), high-velocity air fuel (HVAF), detonation gun (D-Gun) and cold spraying. On the other hand, the processes with the lowest spray speeds are flame spray and electric arc [1,3]. The choice of spraying process depends on the application material's characteristics, the coating's performance requirements, the cost, and the size of the part to be coated [3,7].

Although TS is considered a low-cost process compared to other coating application processes [8], the intensive energy required to heat the application material until it is in a melted state, the gases and the atomising jets used to accelerate the melted or semi-melted particles have a not insignificant impact from an environmental and economic point of view [1,3]. For this reason, the industry that adopts this treatment can face pressure from environmentalists due to the high consumption of chemical substances, the emission of gases into the atmosphere, energy consumption and the generation of hazardous waste from particulate material that has not adhered to the surface of the parts [1,9]. According to Hauschild et al. [10], there are possible environmental risks caused by the operation of TS, and it is necessary for the process to be in constant compliance with environmental laws, in addition to analysing factors associated with the cost of material and energy resources, to analyse the sustainable issues of this process.

In the literature, the analysis of sustainability factors in processes for obtaining surface coatings is generally only carried out using approaches that consider the environmental impacts of each stage, such as Life Cycle Analysis - LCA [11]. This approach can quantify environmental impacts to evaluate and compare the environmental performance of processes and point to the alternative that best meets the required environmental aspects [12,13]. In this scenario, most studies that in some way address sustainability concepts in TS [[11], [12], [13]] are limited to quantifying environmental impacts. In few cases do the existing studies address the costs associated with the process, a part to be considered in the sustainability tripod [14]. This justifies the novelty of this work since it set out to analyse economic and environmental issues in a single approach.

In this sense, this study used the concept of eco-efficiency (EE) to evaluate TS processes, EE being known as one of the main criteria used to assess the ecological performance of organisations [15]. It is considered an improved way of assessing sustainability by relating environmental impacts to economic performance [16], making it a valuable tool for sustainable development [17]. The term “eco-efficiency” was first proposed in 1990 by Schmalleger and Sturm [18]. However, it was in 1992 that the World Business Council for Sustainable Development (WBCSD) introduced one of the main concepts of EE worldwide. EE was defined as the provision of economically competitive goods and services that meet society's needs in terms of quality of life if the entire manufacturing and consumption process (i.e., the life cycle) has the least possible impact on the environment [19].

EE has emerged as a practical decision-support tool by integrating environmental and economic performance [20]. In the scientific literature, there are different EE assessments in agriculture [21], in the food industries [22,23], in the cement industries [24] and in many other industrial and service sectors [25], considering different company sizes [26]. In the context of EE, a process with a low environmental impact may not be economically viable and, consequently, not eco-efficient. Similarly, a process may be economically viable but have a high environmental impact, making it not eco-efficient [27]. Therefore, it is necessary to measure the EE of the process, service or product using the two dimensions of sustainability, economic and environmental, making it possible to understand the value of the process gain in relation to its environmental influence [19].

With environmental impacts being assessed based on indicators that integrate a relationship between consumption and production in economic activity, the use of eco-indicators becomes an important strategy for assessing EE in a simple way since they provide a standardised and quantifiable way of measuring environmental performance [28]. These eco-indicators can be used in a model developed specifically to assess EE in the desired area. In addition, these can be used in decision-making processes to support managerial and strategic decisions [29].

When analysed in organisations, obtaining a company's EE report aims to assess how efficiently resources are used and how effectively environmental impacts are minimised across a company's operations. In other words, it provides information on the company's environmental performance in relation to its financial performance. EE information complements financial and environmental statements to improve company performance from three perspectives [30]: first, EE increases a company's competitiveness in the marketplace since environmentally friendly products and services attract consumers, and companies that prioritise EE can differentiate themselves and attract environmentally conscious customers; second, EE helps organisations assess their compliance with environmental regulations and standards to identify areas where improvements are needed; and thirdly, EE practices can improve engagement with stakeholders, including customers, employees, investors and communities, to increase the company's trust and credibility with its stakeholders.

In line with these impacts, evaluation models with EE indicators can provide companies with a way of becoming more competitive, focusing on innovation, and analysing the sustainability tripod's two pillars (environmental and economic). The development and application of EE models can also indicate improvements in the analysed process, reducing environmental burdens and adverse effects on the environment and human health and increasing process efficiency. EE assessment can help a company identify the appropriate strategy for improving efficiency, guaranteeing sustainable development [31].

In the specific case of this research, it is essential to evaluate the two pillars of sustainability in TS processes, the focus of this study, applying models and approaches that can provide an overview of this scenario. A systematic literature review conducted by Belem et al. [32] pointed out the absence of an EE evaluation model for TS processes, indicating a direction for this study. In addition, the literature review highlights the lack of studies on the practical implications of EE in the company. Currently, most scientific studies on EE are focused on theoretical approaches, in which the results arising from practical improvements have not been adequately evaluated [30]. Similarly, the potential contributions of EE to industrial sectors are not investigated, and the benefits of this approach are often underestimated or overlooked [33]. This article proposes a model with EE indicators to fill these gaps in line with these concerns. The model approaches a practical process to provide new insights, improve current practices, and serve as a framework for evaluating EE in industrial processes. The model implemented in TS processes can enable a more assertive evaluation for the company regarding the reduction of materials, energy, resources and costs.

This study raised the following research question: how can the thermal spray process be evaluated from the point of view of eco-efficiency, and what indicators can be used for this evaluation?

This article proposes a conceptual model to evaluate the EE of TS treatment, selecting environmental and economic indicators considered more impactful in the process.

This paper is structured as follows: section 2 presents a contextualisation of TS processes; in Section 3, the concept of EE and the corresponding indicators are introduced; Section 4 provides the methodology used to achieve the defined objective, the proposed conceptual model for evaluating the EE of TS processes are presented in section 5. A numeric simulation of the conceptual model application is presented in section 6. Finally, conclusions and future development are suggested in section 7.

## 2 Thermal spraying coating processes

TS is a manufacturing process for making resistant coatings on metal parts subjected to different aggressive agents. Spraying materials can be used as wire, powder, and strands. It is possible to use metal alloys, ceramics, and polymers to apply and form protective coatings. TS is defined as a group of processes in which metallic or non-metallic materials are deposited in a melted or semi-melted condition on a previously prepared substrate, forming a coating [34].

Technological advancement led to the development of new and improved equipment and materials for TS, creating numerous opportunities within surface engineering. It is a cost-effective method to prepare coatings widely used in various sectors, such as aerospace, automotive, electrical, and electronic industry, medical applications, paper and pulp, petroleum and chemical industries, ceramic and glass manufacturing and other processes for corrosion and wears protection [1]. Grand View Research (GVR) points out that the global thermal spray-applied coatings market was valued at $10.73 billion in 2021 and is expected to grow by 4.8 % between the years 2022 and 2030 [35].

There are different TS techniques, which can be differentiated from the way of generating energy in the spray equipment to heat the particles, which can occur by adopting combustion gases (Flame Spray, High-Velocity Oxy-Fuel Coating – HVOF and Detonation spraying - D-gun) or electric arc (Wire Arc, Plasma and Plasma Transferred Arc – PTA) [2,34]. Each process consumes a type of material that can be in the form of powder, wire, or strand. The amount of raw material consumed varies for each process, affecting the corresponding impact in environmental and economic terms [11].

In TS, it is important to consider the optimal combination of the material to be used with the selection of the spray gun and its equipment for a specific application. The optimised combination reduces the energy, raw material, and application time in obtaining coated parts [36].

The aspects present in the spraying process and in the coating (resulting product) must be analysed and discussed in relation to the environmental impacts. Consistent with this end, the TS processes require proper procedures to minimise energy, materials, and water consumption. In addition, it is necessary to ensure that the process does not generate ecological damage. If it is not possible, the impacts should be minimised by reducing dust residue, radiation, and noise, consistent with the current legislation. Similarly, the sprayed coating must be free of materials harmful to the environment, human health, and safety [37].

**Fig. 1:**
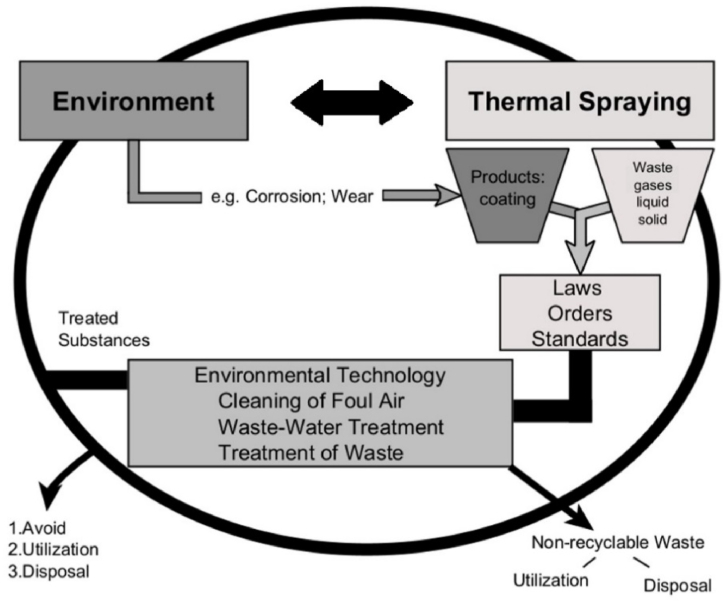
Environmental relationships of the TS process. Source: Adapted from Steinhäuser et al. [37].

At each stage of the TS process, as with any other surface coating application technology, there are ‘contact points' with the environment, as shown in Fig. 1.

The ‘points of contact’ mentioned by Steinhäuser et al. [37] refer to the interactions that exist between materials (metallic, ceramic, polymeric) with the environment, requiring the use of a technology capable of protecting materials against corrosion and wear caused by contact with the environment. Surface techniques, such as thermal spraying, are a viable alternative to produce protective coatings, generating numerous contacts with the environment and providing products while generating waste from the process.

Still, the parameters affecting the environmental impact of TS processes were raised by Serres et al. [11]. These parameters must be considered if the study of the spraying process' impact on the environment is needed. These impacts are atmospheric emissions (gases), solid waste generation (particulates from the raw material used in the deposition), and the process's efficiency, which can increase all consumption due to gas, application material and electricity. Evaluating these sources of environmental impact can provide insight to improve the process, aiming to avoid or eliminate the adverse effects on the environment and human health generated during the deposition of the coating.

All coating application processes generate impacts on the environment and human health. Therefore, the importance of quantifying, for each process, the environmental impacts resulting from the use of material and energy resources, as well as atmospheric emissions and waste generated, must be emphasised.

The comparison of the environmental impacts of different coating processes is considered by Moign et al. [13] as a difficult task due to the number of parameters that each technique includes, which can influence the result. The authors showed that TS processes could significantly affect environmental impacts compared to electroplating processes, which generate large amounts of gas and water emissions due to the use of different chemicals. Therefore, it is fundamental to identify the environmental impacts of the different coating application processes, facilitate comparative analyses and choose the less aggressive process for the environment.

Consistent with the overview of the TS processes presented, it can be inferred that this process is important for the different industrial sectors that need protective coatings to ensure an increase in the useful life of their equipment, machines, or industrial parts. Furthermore, it was noted that such a coating application process includes several characteristics and parameters to be considered when the process's two sustainable aspects (environmental factors associated with economic factors) are analysed. These factors are directly associated with the resources consumed (raw material and energy), the environmental emissions (pollutant gases into the atmosphere and soil), and the safety and risks associated with human health. The EE concept was used to develop an evaluation model adopting the appropriate indicators to assess the performance of TS processes in economic and environmental terms.

## 3 Eco-efficiency

Eco-efficiency combines economic and environmental factors to evaluate a given system, relating the indicators in a structure that can be applied in many different areas. In 2006, the WBCSD presented EE as a fundamental equation in which the numerator identifies the value of the product or service produced and the denominator is represented by the environmental impact caused by the activities performed, as shown in equation (1).

(1) $$Eco-efficiency=\frac{Product\;or\;service\;value}{\;Environmental\;impact}$$

This EE equation can be applied in different systems, so the data selection that will make up the economic and environmental indicators depends on the aspects considered necessary by decision-makers [38].

EE can be applied in the business context to evaluate the product, process, corporation, or sector level [39,40], as well as to evaluate cities, regions, and countries [[41], [42], [43], [44]]. Six strategies proposed by the WBCSD [19] lead a company to become more eco-efficient; they consist of reducing the amount of material used, reducing the intensity of energy used, increasing recyclability, maximising the use of renewable resources, increasing product durability, and maximising service intensity. Therefore, organisations are expected to offer goods and services that use the least resources, generate the least amount of waste and other pollutants, and provide the maximum value [38].

Organisations can see EE as an environmental performance indicator or even a business strategy aimed at sustainable development. There are four actions associated with EE definitions: produce more with less, produce more added value with less environmental impact, use this sustainable concept as a management strategy, and guide the organisation towards sustainable issues [45].

Recent scientific studies show that in the EE evaluation of a specific system, it is necessary to select approaches, methods, or models consistent with the target to be assessed, integrating appropriate indicators and indexes according to the boundary delimitation of the system to be investigated. For instance, the application of approaches such as life cycle analysis (LCA) to identify environmental aspects and data envelopment analysis (DEA), which incorporates different inputs and outputs in different dimensions (environmental and economic indicators), to assess the EE models [46,47]. The development and application of EE models can guarantee improvements in the analysed process, pointing to the reduction of environmental loads and adverse effects on the environment and human health while increasing the efficiency of the process. From the EE evaluation, the applied model can assist the company in determining the best strategy to ensure efficiency and sustainable development [31]. Consistent with a recently published systematic literature review, a research gap exists in the EE evaluation for TS processes [32]. The proposed study aims to fill this gap by adopting a new conceptual model.

## 4 Methodology and systems’ boundaries

**Fig. 2:**
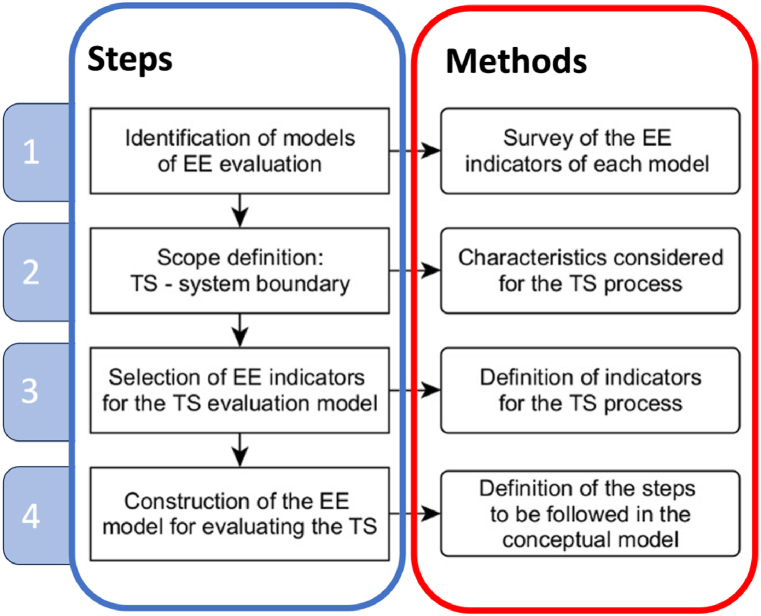
Steps followed in developing the conceptual model for EE evaluation.

Four stages were established to achieve the objective proposed in this study, and the respective methods were adopted to develop the conceptual eco-efficiency evaluation model for thermal spraying processes. The methodology used and its steps are summarised in Fig. 2.

The first stage involves identifying the existing EE models by adopting a recent Systematic Literature Review [32]. In this study, forty articles referred to EE models were analysed. The collected papers aiming to evaluate processes, products, or services in terms of EE were classified into nine areas according to their application sector (i.e., electrical and electro-electronic, civil construction, water resources and water treatment, food industry, farming, thermoelectric and thermal power plants, smart cities, mining, and Manufacturing), to make it easier for the interested researcher to choose the evaluation model in relation to the branch of activity that is intended to evaluate EE. The TS processes were included in the “Manufacturing” area; therefore, the EE indicators selected are consistent with this application sector. This approach ensures that all selected indicators meet the TS process features and provides a preliminary identification of the TS system boundaries (second and third stages of the methodology).

**Fig. 3:**
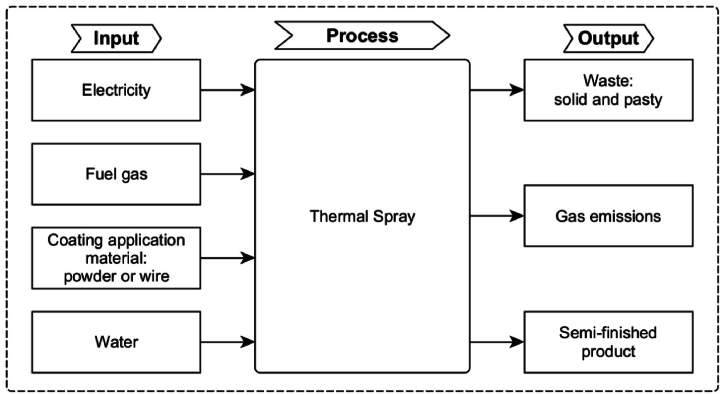
System boundary and input/output flows of the thermal spraying process.

Generally, the TS process consists of preliminary activity to clean the surface followed by abrasive blasting, preheating the surface, applying the material to obtain the coating with the desired thickness, and finishing steps. Nevertheless, in this study, only the factors included in the application phase of the coating on the part were considered. That is, the analysis was limited to the materials used and other products obtained in the phase in which the metallic surface of the part gets the protective coating (semi-finished product). The other phases are not included in the boundary of the study, as they are not the focus of this research. The input and output flows to be considered in the system are summarised in Fig. 3.

In the system, it was considered as input flows the use of energetic resources, such as electricity, fuel, chemicals, amount of application material (in the form of powder or wire) and water. The output flow of the system consists of emissions of gases, solid or pasty residues that remain of the material after the coating application, and the semi-finished product. Generally, other finishing processes are adopted to treat the semi-finished product; these treatments are not included in the systems’ boundaries.

The system limits described above were considered for the most common TS processes. It is possible that, in the case of TS, different variables (in input and/or output flows), depending on the specific process, should be considered. For instance, some processes use combustion gases to heat the particles that will be applied to the coating, while others use different energetic resources. Still, each process consumes a type of material that can be in different forms and presents different temperatures and speeds. Possible adjustments to the general system limits should be evaluated for each TS technique according to the analysis of the indicators in the model application.

## 5 Description of the conceptual model

**Fig. 4:**
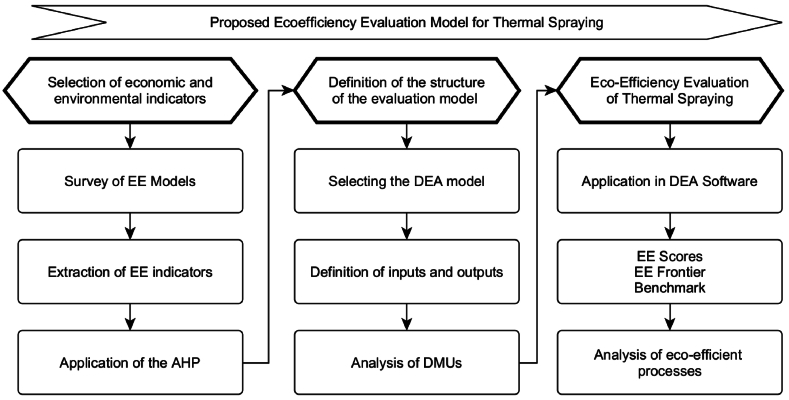
The conceptual model for evaluating EE for thermal spraying processes.

The introduced conceptual model starts with identifying the input and output indicators (environmental and economic), defining the structure of the model, and assessing the EE by applying the DEA model, using software to obtain and analyse the EE of the process. As a result, the steps of the conceptual model of EE evaluation for the TS can be summarised, as shown in Fig. 4.

### 5.1 Selection of economic and environmental indicators (inputs and outputs)

In this first step, the EE indicators were extracted from each existing model included in the “manufacturing” area to evaluate the EE of the TS processes. This approach allowed to identify the fifteen indicators to be considered for TS processes (Table 1).

**Table 1:** Indicators (criteria) to evaluate in the AHP, extracted from the Manufacturing sector.

EE Indicators	Description	Reference
COE	CO_2_ Emission	[48,49]
WCO	Water consumption	[48]
ECO	Energy consumption	[40,48,49]
GHG	GHG Emissions	[48,49]
CFP	Carbon Footprint	[50]
WGE	Waste Generation	[48,49]
MRC	Material resources consumed by the equipment	[40]
CP-HR	Cost of production (human resources)	[49,50]
TEP	Total Economic Production	[48]
QPR	Quantity of pieces (coated piece)	[40]
RMC	Raw material cost	[51]
MCP	Manufacturing cost (process)	[51]
TPC	Total product cost (value)	[52]
WMC	Waste management cost	[51]
TCO	Transportation Cost	[51]

Once this analysis was finished, a survey analysis of all EE indicators (environmental and economic) present in each model was conducted. This step ensured that all extracted EE indicators were judged by adopting the AHP method. Table 1 presents the list of fifteen indicators extracted from the models of the articles only on the “Manufacturing” sector, presented previously by Belem et al. [32].

**Fig. 5:**
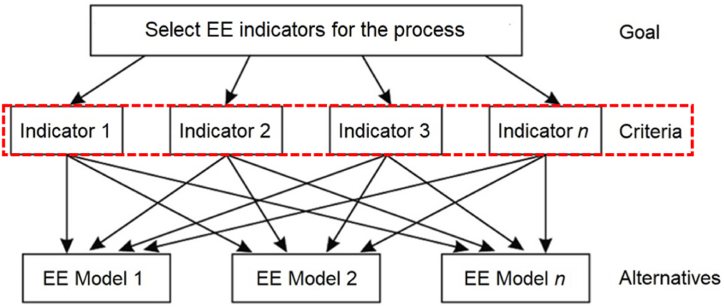
AHP structure for selecting EE indicators for TS processes.

The first step in applying the AHP consists of defining the hierarchy of the decision-making process [53] (Fig. 5): at the first level, the decision objective is defined as “Select EE indicators for the process”; the second level of the hierarchy concerns the criteria identified as most appropriate for selecting the EE model, i.e., the EE indicators included in the “manufacturing” area. Finally, the models involving the selected EE indicators are shown at the third level of the hierarchy.

Then, judgments concerning the relative importance between the two criteria and the corresponding priorities (normalised weights) were estimated. It is worth emphasising that the objective consisted of identifying the proper EE indicators for the scenario under investigation. Consistent with this end, experts in the TS processes have been consulted to provide the judgement so that each indicator's “pairwise” judgment is more assertive. Weights for each analysis have been assigned consistent with the importance of the indicators in the TS process, according to Saaty's pairwise comparison scale [54]. The correctness of the AHP application was tested by analysing the order of the Square Matrix and the random index (RI) value according to the random index or average RI of the AHP [53,54]. Finally, the EE indicators about corresponding relevance in TS treatments have been selected in accordance with the weight estimated. This step is finalised when the EE indicators consistent with the TS process to be investigated are chosen.

### 5.2 Defining the structure of the evaluation model

The structure of the EE evaluation was defined by adopting the DEA technique to assess the EE, integrating the economic and environmental indicators (input and output) included in TS treatments.

**Fig. 6:**
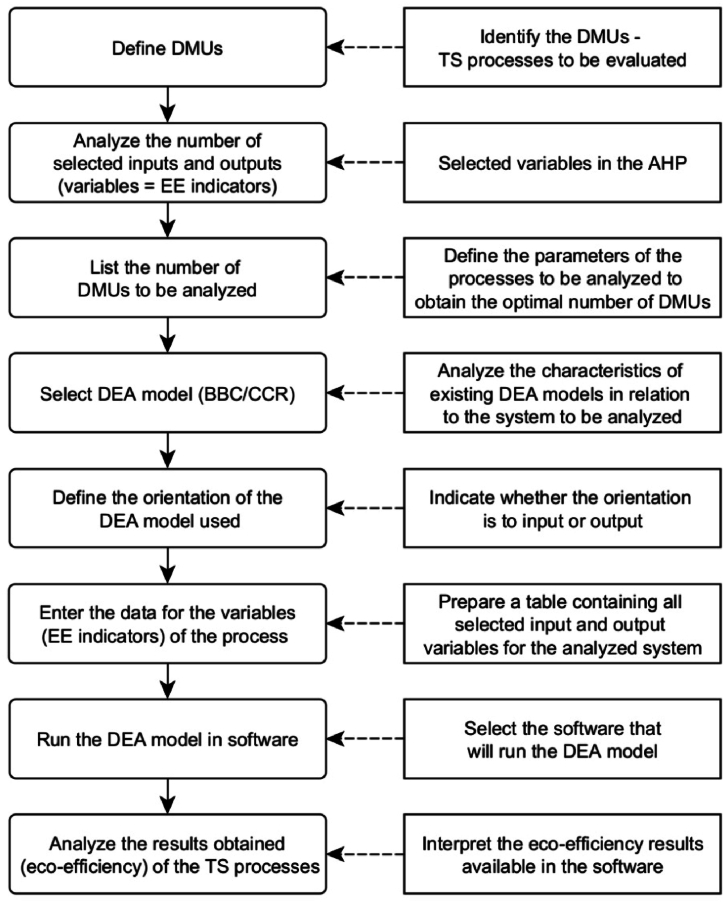
Steps to be followed for DEA modelling (EE evaluation).

DEA consists of the following steps to assess EE, as summarised in Fig. 6:

• To Define the decision-making units (DMUs) that will be compared.
• To analyse the input and output variables so that it is possible to list the number of DMUs to be evaluated.
• To select the model between Banker, Charnes, Cooper (BCC) or Variable Returns to Scale (VRS) and Charnes, Cooper, Rhodes (CCR) or Constant Returns To Scale (CRS). The BCC or VRS models consider scale variation and do not consider proportionality between inputs and outputs. On the contrary, the CCR or CRS models assume constant returns to scale [55].
•To define the DEA orientation (input or output) and identify the variables to be assumed. This analysis must be done by the process specialist, by the company involved in the study, and even by the acting researcher since it is needed that the model's orientation must be aligned with the context and the target of the analysis [56].
• To Collect data, estimate, and evaluate the results achieved.

The number of DMUs must be at least three times the number of variables (inputs and outputs) selected. Suppose a low number of DMUs is identified as having a high number of variables. In that case, it is recommended to increase the number of DMUs by adopting finalised strategies to provide the results according to an assertive EE evaluation [57].

As a suggestion to facilitate the identification of DMUs, it is necessary to consider a set of DMUs that perform the same functions with the same objective, being able to compare these DMUs concerning a certain period (which can be days, months or years, depending on the process analysed) [56].

Once the number of DMUs to be analysed has been identified, the inputs and outputs to be used in the model have been defined, and the DEA model has been identified, the next step consists of collecting data for each indicator and each unit, according to the structure proposed in Table 2. The organisation of variables in table form is essential since it makes it easier for the researcher to group the information and start data processing.

**Table 2:** Distribution of DMUs and respective EE indicators for use in data collection.

Relation: EE Indicators - DMUs										
DMUs	EE Indicators									
	Ind._1_	Ind._2_	Ind._3_	Ind._4_	Ind._5_	Ind._6_	Ind._7_	Ind._8_	Ind._9_	Ind. n
DMU_1_										
DMU_2_										
DMU_3_										
DMU_4_										
DMU_5_										
DMU_6_										
DMU n										

### 5.3 Process eco-efficiency assessment - DEA software

The EE for each DMU was estimated by processing the data adopting the DEA methodology. Several software and web applications are on the market to support this phase [58]. In addition to commercial software, it is possible to conduct DEA analysis using the R programming language. R is free, open-source and highly customisable, allowing the integration of the DEA models with other techniques to support the decision-making process [59].

Generally, the software's choice is independent of the previous steps since all the system's necessary data (DMUs, variables) to be evaluated must be imported according to the structure proposed in Table 2. In this way, the inputs required to use the software are imported, and the outputs will be achieved after running the model can be provided for each unit analysed.

The following results are expected [56]: EE scores of the TS processes, the construction of the eco-efficient frontier, and the benchmark analysis. The EE scores are needed to identify which TS process(es) are considered eco-efficient. The EE score is included in a range between 0 and 1. The DMUs referred to as TS processes with an EE score equal to 1 are considered eco-efficient. On the contrary, DMUs with an EE score of less than one are considered not eco-efficient. Therefore, if an EE score of 0.9 is obtained for a DMU adopting an input-oriented model, this means that the DMU could have a 10 % reduction in its resource consumption, keeping its output constant. Similarly, the EE score of 0.9 indicates that increasing its outputs by 10 % is possible, keeping the same consumptions in the system's input.

Consistent with this view, the DMUs most eco-efficient will be located on the line representing the frontier. On the contrary, the DMUs less eco-efficient will be positioned below the frontier, ensuring, in this way, an optimal relationship between inputs and outputs.

The benchmark analysis is needed to identify the most eco-efficient DMUs and use them as a reference for DMUs that are not considered eco-efficient. According to this approach, the non-eco-efficient DMU uses the eco-efficient DMU variables as a basis to improve its parameters and become eco-efficient.

## 6 Numerical simulation: application of the conceptual model

The model proposed was applied in a numerical case study; all the steps described in Fig. 3 were simulated to evaluate the EE of the TS processes. For the first part of the model, the inputs and outputs of the process under study are selected for later application in the AHP method. In this case, this extraction was performed using the indicators presented in Table 1. Conceptually, the fifteen indicators were arranged in a matrix, and the judgment was performed using Saaty's scale. This verbal judgment refers to the following numerical value [54]: “Extremely important” (9 or 8); “Very strongly more important” (7 or 6); “Strongly more important” (5 or 4); “Moderately more important"(3 or 2); and “Equally important” (1).

To better understand this judgment, the comparison between COE and WCO indicators (Table 3) shows that the WCO indicator received a weight equal to 4 (i.e., “Strongly more important”) than the COE indicator, which gets the inversely proportional value = 1/4, according to the Saaty scale. The same approach was applied to each pair of EE indicators analysed.

**Table 3:** Result of the judgment of the EE indicators through the AHP method.

Criteria (EE Indicators)	COE	WCO	ECO	GHG	CFP	WGE	MRC	CP-HR	TEP	QPR	RMC	MCP	TPC	WMC	TCO	**Σ** $\left(C_{i}\right)$
COE	1	1/4	2	1	1	2	2	3	2	3	2	2	1/2	1/4	2	24.00
WCO	4	1	1	2	2	3	3	3	2	3	4	3	1/3	2	3	36.33
ECO	1/2	1	1	3	4	1/2	3	3	3	3	3	3	3	3	1/3	34.33
GHG	1	1/2	1/3	1	1	1	3	4	2	2	2	3	1/2	3	3	27.33
CFP	1	1/2	1/4	1	1	2	2	3	3	3	2	3	1/2	1	2	25.25
WGE	1/2	1/3	2	1	1/2	1	2	1/2	1/2	2	3	2	1/4	2	1	18.58
MRC	1/2	1/3	1/3	1/3	1/2	1/2	1	3	2	2	1/2	1/2	1/6	1/2	1/2	12.67
CP-HR	1/3	1/3	1/3	1/4	1/3	2	1/3	1	1	1/2	1/2	1	1/7	1/2	1	9.56
TEP	1/2	1/2	1/3	1/2	1/3	2	1/2	1	1	1/4	1	1	1/5	1/2	1	10.62
QPR	1/3	1/3	1/3	1/2	1/3	1/2	1/2	2	4	1	1	1	1/5	1/3	1/3	12.70
RMC	1/2	1/4	1/3	1/2	1/2	1/3	2	2	1	1	1	1	1/4	1/4	1	11.92
MCP	1/2	1/3	1/3	1/3	1/3	1/2	2	1	1	1	1	1	1/4	1/3	1	10.92
TPC	2	3	1/3	2	2	4	6	7	5	5	4	4	1	2	2	49.33
WMC	4	1/2	1/3	1/3	1	1/2	2	2	2	3	4	3	1/2	1	2	26.17
TCO	1/2	1/3	3	1/3	1/2	1	2	1	1	3	1	1	1/2	1/2	1	16.70

In Table 3, the “pairwise” judgment in the AHP (referring to the importance of pairs of indicators for the TS) identifies the average results. It is possible to observe that the main diagonal consists of values equal to 1. The other values and the corresponding inverse values are in a symmetrical position concerning the main diagonal. The last column represents the total sum (Σ) of the rows of the weights obtained in the trial; it was assumed as weight ($C_{i}$) to analyse the consistency criteria of EE indicators (Table 4).

**Table 4:** Analysis of criteria consistency (EE indicators).

Criteria (EE Indicators)	Weight $C_{i}$	Normalised Weight $C_{in}$	$\lambda_{\max}=\left\{\frac{\left(\bar{D_{i}}*\bar{C_{in}}\right)}{C_{in}}\right\}$	$\lambda_{\max}$ (average)	Random Index (RI)	Consistency Ratio (CR)
COE	24.00	0.074	17.30	17.33	1.59	0.09
WCO	36.33	0.111	17.97			
ECO	34.33	0.105	20.74			
GHG	27.33	0.084	16.74			
CFP	25.25	0.077	16.05			
WGE	18.58	0.057	18.89			
MRC	12.67	0.039	15.44			
CP-HR	9.56	0.029	17.62			
TEP	10.62	0.033	18.18			
QPR	12.70	0.039	15.00			
RMC	11.92	0.037	16.04			
MCP	10.92	0.033	16.60			
TPC	49.33	0.151	16.90			
WMC	26.17	0.080	16.73			
TCO	16.70	0.051	19.69			

To perform the following steps in the AHP method, it is necessary to estimate the Random Index (RI) value, which is tabulated according to the order of the constructed matrix. According to Table 3, we have a matrix of order 15 for this study. According to Saaty [54], for a Square Matrix of order 15, the RI value equals 1.59.

Given the results in Table 3, the Consistency Ratio (CR) was identified according to Equation (2) [32,53]. CR provides the level of consistency, in terms of proportionality and transitivity, of the judgment of EE indicators resulting from AHP [53].

(2) $$CR=\frac{\;CI}{RI}$$

where the Consistency Index (CI) is provided in the judgments (level of importance attributed by the pairwise comparison), its value is identified according to Equation (3) [53]. The RI identifies the values assumed in the case of judgments provided by a generation of purely random judgments. RI depends only on the order of the comparison matrix (Table 3) and can be identified by numerical simulations, in which random judgments are generated on the Saaty scale.

(3) $$CI=\frac{\left(\lambda_{\max}\;–\;n\right)}{\left(n\;-1\right)}$$

Where n identifies the order of the comparison matrix. The parameter $\lambda_{\max}$ represents the maximum value of the comparison matrix [53]. It is the average rate (evaluated with respect to n) between the product of the vector of each row of the judgment matrix ($\bar{D_{i}})$ and the vector of normalised weights $\bar{(C_{in}})$ on the n component of the vector of normalised weights $\left(C_{in}\right)$ (equation (4)).

(4) λmax={i=1,n}Average{(Di‾*Cin‾)Cin}

The results of the analysis of the consistency of the EE indicators are summarised in Table 4.

The consistency of the judgment should be checked by comparing the CR value with an acceptability threshold value (usually assumed equal to 0.10). If the CR value is higher than 0.10 there is an inconsistency in the matrix filling in relation to the placed value of the Saaty scale; therefore, in these cases, a new judgment should be performed. CR identified in the numerical simulation has a consistency of 0.09 or 9.0 %, which means that the exemplified judgment is consistent.

The next step consists of analysing higher weights, referred to as presented indicators, to select the inputs and outputs that will be applied in the model. For this case, the average weight ($C_{i}$) is 21. Therefore, only the indicators with a weight higher than 21 were selected.

As a result of the first part of the model, seven indicators were selected to be included in the next stage of data collection. They are CO_2_ Emission (COE), Water consumption (WCO), Energy consumption (ECO), GHG Emissions (GHG), Carbon Footprint (CFP), Total product cost (TPC), and Waste management cost (WMC).

In the proposed model's second step, the EE evaluation structure was defined for the numerical case considered, following the steps introduced in the DEA modelling, shown in Fig. 5. For this case, the CRS model and the input orientation were adopted to reduce the resources used (inputs), ensuring the production level (outputs) in the TS processes. The seven indicators were classified as inputs and outputs. The inputs are the resources included in the process, classified as WCO, ECO and TPC. The outputs represent the results of the process, classified as COE, GHG, CFP and WMC.

For this simulation, the DMUs represent the TS processes. In this case, the TS processes for each DMU were not specified since this analysis aims to provide a general overview of the model application. Therefore, illustratively, a number of DMUs equal to 21 was defined, with the idea of identifying a number three times greater in relation to the number of variables (inputs and outputs) given. The values of each DMU, shown in Table 5, were identified according to a numerical simulation in which each indicator's most probable average values have been considered. These “fictitious” values allow us to assess the EE of the process by using SAGEPE software.

**Table 5:** Data for EE indicators for the fictitious DMUs.

Relation: EE Indicators - DMUs							
DMUs	EE Indicators						
	COE	WCO	ECO	GHG	CFP	TPC	WMC
DMU_1_	2	0.6	2.8	0.02	3	100	0.8
DMU_2_	2	0.03	2.8	0.02	5	80	0.9
DMU_3_	3	0.03	3	0.03	4	100	0.6
DMU_4_	2	0.6	2.8	0.02	6	90	1.2
DMU_5_	5	0.8	3	0.08	2	85	6.9
DMU_6_	2	0.8	1.8	0.02	6	60	6
DMU_7_	2	0.6	1.6	0.02	4	60	5
DMU_8_	4	0.05	1.5	0.06	5	60	8
DMU_9_	4	0.06	2.6	0.07	4	52	7
DMU_10_	4	0.06	2.9	0.07	4	52	5.6
DMU_11_	2	0.06	3	0.02	2	38	4.5
DMU_12_	9	0.6	3.5	0.12	2	39	1.2
DMU_13_	6	0.06	2.8	0.1	3	34	1.9
DMU_14_	4	0.6	2.9	0.1	6	36	6.5
DMU_15_	2	0.5	4	0.02	9	22	6.2
DMU_16_	2	0.5	3.8	0.02	8	110	4.5
DMU_17_	9	0.5	3.5	0.2	2	112	4.6
DMU_18_	5	0.3	3.6	0.22	2	90	1.6
DMU_19_	6	0.5	3.1	0.022	1	98	1.3
DMU_20_	3	0.6	2	0.022	2	94	2
DMU_21_	3	0.6	1.8	0.3	1	96	7

The data in Table 5 were arranged in an Excel spreadsheet to be imported into the SAGEPE software; within this program, the input-oriented CRS model was selected, and the inputs and outputs for processing and obtaining the results were indicated.

**Fig. 7:**
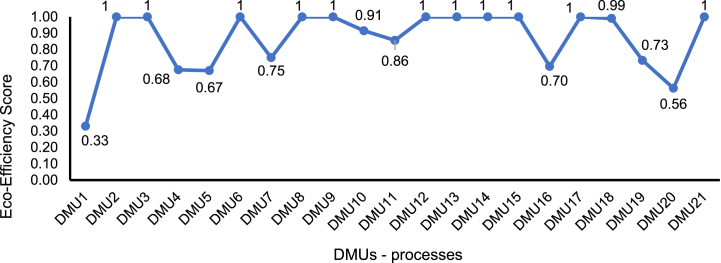
EE score for the DMUs analysed in the simulation of the EE model developed.

The EE results of the DMUs analysed can be easily interpreted in the software, which provides all EE values, the ‘benchmarking’ and the ‘gains’ obtained from each DMU. In the case study proposed, the DMUs with an EE score equal to 1 were DMU2, DMU3, DMU6, DMU8, DMU9, DMU12, DMU13, DMU14, DMU15, DMU17, DMU21, as shown in Fig. 7.

Among 21 DMUs investigated in this numerical simulation, 11 are eco-efficient since they showed an optimal relationship between their inputs and outputs and can serve as benchmarks for the other DMUs.

In the numerical case, the lower EE scores were identified for DMUs with numbers 1, 16, and 20. This occurred because the values of the EE indicators of these DMUs did not present an ideal combination to be considered eco-efficient. In the DEA software used in this case (i.e., SAGEPE), the weights for each indicator are calculated automatically, according to the DEA model; in other words, there are no restrictions and changes in the weights by the user. The DMUs assessed as “not eco-efficient” require further details, especially in the analysis of the gain. This analysis allows us to investigate the resources to be reduced consistently with each indicator to increase the EE score of less performing DMU.

The EE analyses were not deepened for the case presented since the objective was only to describe the approach introduced with a numerical case study, presenting the results for each step of the proposed model. However, from the results obtained in the software, all the data should be interpreted to discuss the relationships between the inputs and outputs of the DMUs and facilitate decision-making about improving the processes under study, considering the benchmarking presented.

The results highlighted the effectiveness of the developed model in assessing EE due to TS processes. The model has provided an evaluation consistent with the existing studies; this is because the construction and arrangement in sequential steps, aligned with applications of approaches consolidated in the literature, such as AHP and DEA, have provided an assessment of the environmental and economic performance of the process.

The combination of approaches and methods to ensure a more efficient and effective decision-making process has been adopted by other researchers [60]. Other studies have shown the evaluation of EE with steps to be followed, combining DEA with different approaches such as LCA + DEA [[61], [62], [63]] or using the carbon footprint, known as the CFP + DEA method [64,65]. In both cases, the target approach integrated with the DEA models allowed the evaluation of the process's impact on given variables. On the contrary, in this study, applying AHP allowed experts to judge which variables are the most appropriate for the EE evaluation using DEA.

However, from a conceptual point of view, the model proposed in this study has the advantage of following sequential stages that lead to the inclusion of environmental and economic variables in TS processes. From a practical point of view, the selection of variables by using the AHP and then obtaining the data following the recommendations described facilitates the researcher in applying the method and achieving EE results to be assessed. This approach can be considered new and challenging to compare with similar works, generally focused on a pre-defined set of given variables.

## 7 Conclusion

TS is a coating application process that consumes energy, other resources, and materials. It results in important protective coatings for industries from various sectors that need their parts and/or machinery to have adequate protection to increase the service life and, consequently, improve quality and reduce costs. The study showed that optimising these processes is an opportunity to ensure that industries become increasingly aware of the environmental impacts that their operations cause to society.

EE, as an analysis from the point of view of two aspects of sustainability, is a way to be applied within this sector since there is a lack of research involving both environmental and economic aspects in a single approach. Thus, this study has achieved the objective of proposing a model composed of steps to be followed to evaluate the EE of coating application processes.

The first part of the proposed model uses the AHP method, considering the EE indicators surveyed in the literature, to select the economic and environmental indicators. The values of these indicators are collected for each DMU in the second part and introduced in a DEA model according to the purpose intended to be pursued. Finally, in the third part of the proposed model, it is possible to evaluate the EE values by adopting the software of the selected DEA model and making available the EE results to be analysed for the research manager and/or the specialists involved.

With the numerical simulation performed in section 6 of this paper, it was possible to validate the model, following all the steps proposed in the qualitative (conceptual) framework seen in Fig. 4. The example presented here is considered an important contribution for researchers who want a starting point for evaluating EE in their intended context. Thus, the analysis and data collection that must be performed to integrate the AHP and DEA in the proposed model was discussed, and the result of the EE evaluation was obtained.

The limitations of the research can be classified according to three perspectives:

1. The steps to identify the value of EE indicators were not deepened since the purpose of the research conducted consists of describing the model and its application for TS processes, using EE-values already known in scientific literature. In other words, the EE model doesn't allow estimating EE indicators but suggests a methodology to adopt them in an industrial application.
2. The results obtained by SAGEPE software were limited to analysing the EE scores, not exploring the benchmark and gaining in-depth results, which is necessary to understand why certain DMUs presented a low EE.
3. The investigation is related to only a phase of the TS process (i.e., coating of the part). According to experts, this is the most impactful phase from an environmental and economic perspective. Nevertheless, it should be very interesting to extend the analysis to other phases of the treatment to assess their contribution to the overall impact of the entire process.
4. The proposed research work points out an indication for assessing EE but does not provide a guideline for the company on how to improve EE.

Consistent with the raised open issue, it is suggested, as future research, to apply the model presented in this paper to assess the EE of TS processes according to three strategies. The process should be analysed, starting with the identification of EE indicators; all phases of the treatment should be considered, and insights on the possible strategy to improve EE performance should be provided.

## CRediT authorship contribution statement

**Maria Julia Xavier Belem:** Writing – review & editing, Writing – original draft, Methodology, Investigation, Data curation, Conceptualization, Formal analysis, Software, Validation. **Milton Vieira Junior:** Writing – original draft, Validation, Supervision, Methodology, Conceptualization, Formal analysis, Investigation. **Giovanni Mummolo:** Supervision, Conceptualization, Formal analysis, Investigation, Methodology, Validation. **Francesco Facchini:** Writing – review & editing, Writing – original draft, Supervision, Conceptualization, Investigation, Resources, Visualization.

## Declaration of competing interest

The authors declare that they have no known competing financial interests or personal relationships that could have appeared to influence the work reported in this paper.
